# Analysis of COVID-19 Spread in Tokyo through an Agent-Based Model with Data Assimilation

**DOI:** 10.3390/jcm11092401

**Published:** 2022-04-25

**Authors:** Chang Sun, Serge Richard, Takemasa Miyoshi, Naohiro Tsuzu

**Affiliations:** 1 Data Assimilation Research Team, RIKEN Center for Computational Science (R-CCS), Kobe 650-0047, Japan; chang.sun@a.riken.jp (C.S.); takemasa.miyoshi@riken.jp (T.M.); naohiro.tsuzu@riken.jp (N.T.); 2 School of Science, Nagoya University, Chikusa-ku, Nagoya 464-8602, Japan; 3 Graduate School of Mathematics, Nagoya University, Chikusa-ku, Nagoya 464-8602, Japan; 4 Prediction Science Laboratory, RIKEN Cluster for Pioneering Research (CPR), Kobe 650-0047, Japan; 5 RIKEN Interdisciplinary Theoretical and Mathematical Sciences Program (iTHEMS), Wako 351-0198, Japan

**Keywords:** agent-based model, data assimilation, COVID-19, effective reproduction number, 92D30

## Abstract

In this paper, we introduce an agent-based model together with a particle filter approach to study the spread of COVID-19. Investigations are mainly performed on the metropolis of Tokyo, but other prefectures of Japan are also briefly surveyed. A novel method for evaluating the effective reproduction number is one of the main outcomes of our approach. Other unknown parameters are also evaluated. Uncertain quantities, such as, for example, the probability that an infected agent develops symptoms, are tested and discussed, and the stability of our computations is examined. Detailed explanations are provided for the model and for the assimilation process.

## 1. Introduction

The outbreak of COVID-19 had a huge impact on human society, and has also triggered an enormous scientific response. In this paper, we provide a rather detailed account of an agent-based model for the estimation of the effective reproduction number $R_{t}$. Our approach is based on data assimilation and on a particle filter scheme.

The effective reproduction number is one quantity of major importance for any epidemiological studies, as indicated in the studies [1,2,3,4,5] and references therein. Since it works as an estimator of the number of secondary cases produced by a primary case at time *t*, it provides crucial information about the spread of the disease, allowing health services to evaluate previous interventions and forecast the evolution of the epidemic. However, its precise evaluation is very challenging, and several independent approaches exist or have been recently developed. As thoroughly investigated in [6,7], all these techniques present advantages and flaws, but nevertheless each of them brings some information.

One of the earliest attempts to implement epidemiological observations with epidemic models through a variational data assimilation approach was published in [8]. In relation to COVID-19, by March 2020, some investigations using a method called Ensemble Smoother with Multiple Data Assimilation (ES-MDA) appeared in a preprint format [9], quickly followed by [10], which forecasts the evolution of the epidemic in Pakistan with a Kalman filter coupled with the Arima model. Still, in the early responses, let us mention the implementation of a switching Kalman filter [11] to overtake the Gaussian and linearity assumptions, while an extended Kalman filter is used in [12] for an evaluation of the effective reproduction number. However, as acknowledged by the authors in [12] “the presented approach can only be used for countries/regions/areas that have performed mass testing with laboratory confirmation”, which drastically reduces its applicability. In another direction, let us mention [13] where a Kalman filter and random forest algorithms are applied to make short-term predictions in India.

Another important work in this framework is the international collaboration [14], in which it clearly presents that the regional characters of some parameters are important. More recently, let us mention [15] in which a model with vaccination and using an Ensemble Kalman Filter is implemented for Saudi Arabia, and [16], which discusses vaccination strategies by using data assimilation techniques. Reference [17] tracks the effective reproduction number with the Kalman Filter, and provides estimates for the effectiveness of non-pharmaceutical interventions, while [18] uses Bayesian sequential data assimilation for forecasting the evolution of COVID-19 in several Mexican localities. Additional recent investigations mixing data assimilation techniques and epidemic models can be found in [19,20,21,22,23,24,25].

In contrast to the references mentioned above, contact-based (or agent-based) models provide a more microscopic approach for the spread of diseases; see, for example, [26]. Up to the best of our knowledge, very few works have investigated the COVID-19 outbreak with such models coupled with epidemiological data. As a rare example, let us mention [27] which uses an ensemble Kalman filter and a undirected graph of 200 nodes with a small-world topology for estimating the evolution of the epidemic.

In the present study, we further develop the agent-based model and the assimilation of data, but simplify the graph structure. More precisely, we propose a method coupling a particle filter with an agent-based model for the evaluation of the effective reproduction number $R_{t}$. It consists of two main steps, which are repeated on a regular basis: (1) Generate a prediction for the model’s state on the next time step based on the current model’s state, and move the time step forward; (2) update the model’s state based on observations on the current time step. Since new data about COVID-19 are available on a daily basis, we regard one day as one time step in our model. In one experiment, numerous independent simulations are considered simultaneously, and consequently some quantities of interest can be estimated with a probability distribution.

We also aim to make our approach and results easily reproducible. Since some parameters in the model are location-dependent, and in order to rely on a constant source of observations, our investigations are mainly focusing on the metropolis of Tokyo. We also provide results for two other Japanese prefectures and the entire country, and these investigations are briefly discussed. The selection of these prefectures is motivated by the diversity of their size, and by the respective impact of the epidemic. This illustrates that our approach can be adapted to different cities, regions, or countries, with little effort.

**Figure 1:**
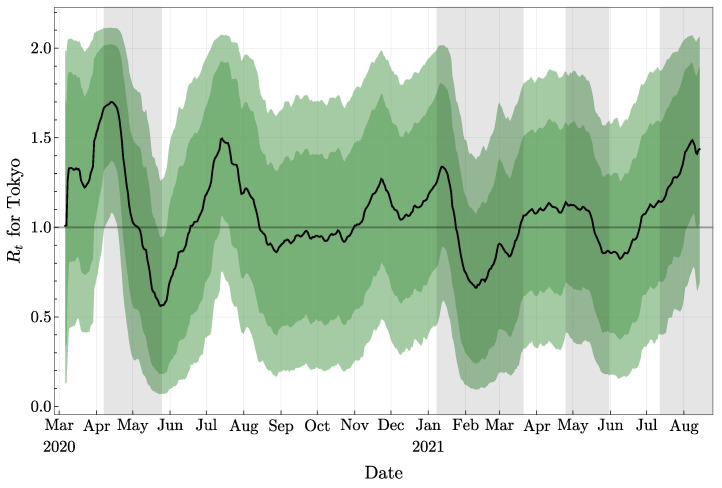
Effective reproduction number. The black curve indicates the mean value obtained from numerous independent simulations; the dark and light colored regions refer to the 68% and 90% confidence intervals, respectively. States of emergency correspond to grey regions.

In Figure 1 we present our main result about the evaluation of the effective reproduction number $R_{t}$ for Tokyo, from 6 March 2020 to 14 August 2021. Our approach provides a daily mean value and confidence intervals based on the distribution of parameters from numerous independent simulations.

Before moving to a more precise description of the content of this paper, let us emphasize a few key points of our investigations. First of all, our approach does not rely on a knowledge of the size of the underlying population. This is in contrast with the standard SIR compartmental model involving differential equations, for which the size of the susceptible population is part of the initial condition. The proportion of fully (or partially) vaccinated persons inside the population is also not a necessary information for our investigations. Accordingly, acquiring a immunity (or not) after recovering from the disease does not play any role in our approach. In fact, these non-dependencies are a special feature of the phenomenological character of our method for the computation of the effective reproduction number. Relatedly, note that our computations does not involve an inverse problem, nor rely on Bayesian estimation, or on any assumption of Poisson distributions. For comparison, let us mention for example [28] which presents an “inverse method for extracting the full transfer function of infection, of which the reproduction number is the integral”, or [29] for a statistical approach for the estimation of the instantaneous reproduction number, involving Bayesian estimation and the assumption of some Poisson distributions.

Let us now describe the content of our paper. In Section 2 we precisely introduce our agent-based compartmental model (an extended version of the SEIR model): its compartments, the flows between these compartments, the probability of agents entering each compartment, and the time agents spend in each compartment. Some of these values are fixed, in which case we provide a reference; other values are random variables, whose distributions are either provided by some references or will be evaluated during the experiments. The procedure for producing secondary cases is also described, and the computation of the effective reproduction number $R_{t}$ is explained.

In Section 3, the evolution process and the data assimilation technique are introduced. The leading idea is to consider simultaneously numerous independent epidemics (called particles), and to associate to each of them a weight on a daily basis. The weights are assigned based on the proximity of the simulated epidemics to the observations. More precisely, since direct observations are only possible for three compartments of the model (the agents under medical treatment, the agents who have recovered after medical treatment, and the deceased agents), we compare the number of agents in these three compartments to the corresponding observations, and compute the weights accordingly. Various plots illustrate the outcomes of our experiments, such as, for example, the total number of asymptomatic agents. A resampling process necessary for our experiments is also introduced and discussed. In the final part of this section, we provide some estimations about two parameters evaluated during the experiments.

A discussion about the probability than an infected agent develop symptoms is presented in Section 4. Indeed, for COVID-19 this probability is still not precisely known, and contradictory values can be found in the literature; see, for example, [30,31]. Similarly, it is not certain by how much asymptomatic agents are less infectious than symptomatic agents, and various numbers can be found in the literature [32]. For this reason, we performed stability tests on our model with different probabilities of developing symptoms, and different relative infectivities, and compared the resulting effective reproduction numbers. At a more technical level, we also discuss in this section the relationship between the stability of the computations and the frequency of resamplings introduced in Section 3.

In Section 5, the last section of this paper, we compare our results for the effective reproduction number with similar estimations from independent sources. In particular, one of these estimations is based on a simplified version of a maximum likelihood estimation for the effective reproduction number provided by H. Nishiura [33]. Additional results for other prefectures and for Japan are provided and discussed. We also expose the strength and the weakness of our approach. Some possible improvements are presented, and future extensions are finally discussed.

## 2. Description of the Model

**Figure 2:**
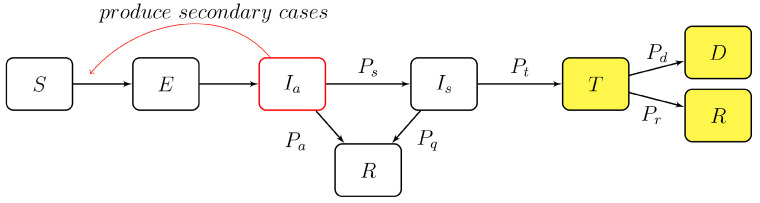
The compartments and the probabilities of each path. Data provided by health authorities are available for the compartments colored in yellow.

The model consists of an extension of the well-known SEIR model, but is adapted in the context of an agent-based model. It is based on seven compartments, as shown in Figure 2, which can be described as follows:

*S*  The compartment of all susceptible agents.
*E*  The exposed agents: right after infections, agents move from *S* to *E*, and they are not infectious in this compartment. They are not recorded by health authorities.
*I_a_*  The asymptomatic infectious compartment: one part of these agents are asymptomatic (will never develop any symptom), while the other part of these agents are pre-symptomatic. The asymptomatic agents are leaving $I_{a}$ after recovery. Pre-symptomatic agents will move to $I_{s}$ as symptoms develop. They are not recorded by health authorities yet. Note that the labels and the definitions used for $I_{a}$ and $I_{s}$ (introduced below) coincide with the ones used in [34], but $I_{a}$ corresponds to the compartment *P* in the SEPIA model, see [35].
*I_s_*  The symptomatic infectious compartment: agents are showing mild or severe symptoms. Most of these agents will look for medical support, but others will self-quarantine without contacting any health authority. The first cohort will move to *T* once supervised by health authorities, while the latter cohort will leave $I_{s}$ after recovery.
*T*  The agents undergoing treatment. These agents are treated in hospitals, at home, or in other facilities. They are recorded by health authorities.
*D*  The deceased agents coming from *T*. They are recorded by health authorities.
*R*  The recovered agents: The agents coming from *T* are recorded by health authorities, while the ones coming from $I_{a}$ or from $I_{s}$ are not recorded by any health authority.

As shown in Figure 2, should a compartment have multiple outflowing paths, its agent will enter different paths following certain probabilities. We provide in Table 1 the different values and the sources. Note that these values will be discussed again with additional experiments in Section 4. Note also that the probabilities $P_{d}$ and $P_{r}$ will be evaluated during the experiments, and therefore are time (≡day) dependent. For the probability $P_{q}$ of self-quarantining without contacting any health authority, since no information is available for Tokyo, we use the result of the survey [36] conducted in Osaka. As emphasized in this survey, it is mainly because of social pressure that some symptomatic persons prefer self-quarantining without contacting any health service.

**Figure 3:**
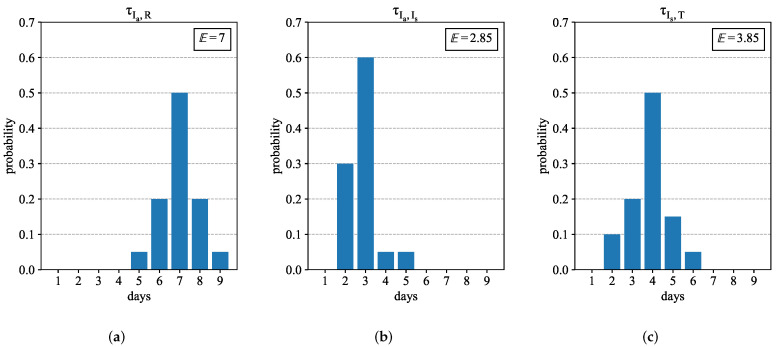
Distributions and mean values for: (**a**) infectious period $\tau_{I_{a},R}$ for asymptomatic in compartment $I_{a}$; (**b**) infectious period $\tau_{I_{a},I_{s}}$ for pre-symptomatic in compartment $I_{a}$; and (**c**) time $\tau_{I_{s},T}$ spent by symptomatic in compartment $I_{s}$ before undergoing treatment.

In some of these compartments (Cpt), the number of days spent by agents is important. We list in Table 2 the necessary information about these durations and provide the sources. Some given distributions are provided in Figure 3. Note also that the time duration $\tau_{T}$ (time in compartment *T*) will be evaluated during the experiments, and therefore is time dependent. Note that if any of the parameters are time dependent, the behavior of agents are determined by the parameters on the day they enter their current compartments.

Let us now emphasize some assumptions made in our model:

(1) Only agents in $I_{a}$ are going to spread the disease. Indeed, asymptomatic agents or pre-symptomatic agents in $I_{a}$ are not aware of their condition but are already infectious. On the other hand, agents in $I_{s}$ are aware of their condition and are supposed to take all precautionary actions for not spreading the disease. Clearly, this is a simplifying assumption, but it is known that the transmission of the virus is mostly taking place before the appearance of the symptoms, with a gradual decline of virus load after the appearance of symptoms [40]. Also, without this assumption one should introduce an additional coefficient reflecting the fraction of symptomatic agents (aware of their status) spreading the disease. Clearly, very little information is available about such a parameter.
(2) Births and natural deaths are not included in the diagram flow of Figure 2. As discussed in Section 5, these agents would not play any role.
(3) The immunization status of agents in the compartment *R* is not specified and does not play any role in our model. This is also discussed in Section 5.

**Figure 4:**
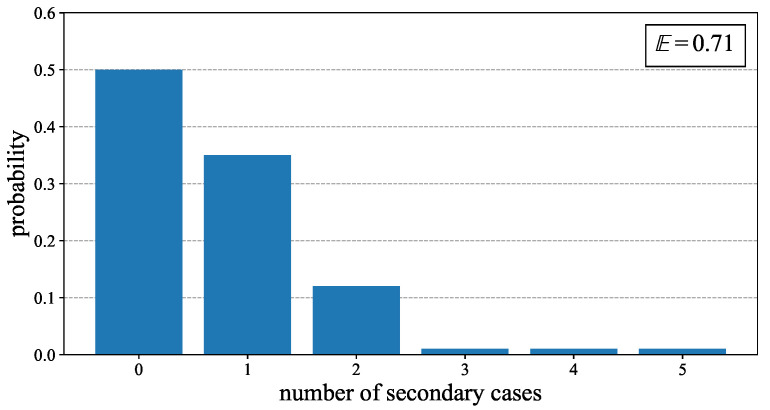
Distribution and mean value of the daily offspring distribution Od.

Thus, for agents in $I_{a}$, a simplified daily offspring distribution Od is provided in Figure 4, and we refer for example to [41] for more details. The implementation of this distribution is explained in the next section.

One important question concerns the relation between the transmission coefficient for asymptomatic agents and the transmission coefficient for pre-symptomatic and symptomatic agents. For our investigations, we shall rely on the result of the systematic review [32]. Consequently, we shall fix this relative infectivity coefficient *k* to 0.58. This factor is slightly smaller but of a comparable scale compared to earlier investigations; see, for example, [30]. This factor will be discussed again with additional experiments in Section 4.

Let us set $r_{t}\in \left[0,1\right]$ as a time-dependent multiplicative factor which takes into account the real interaction between agents. This factor (updated on a daily basis) depends on individual behavior but also on non-pharmaceutical interventions. As a consequence, for asymptomatic agents in $I_{a}$, the daily production of second-generation infections is given by the daily offspring production with all # secondary cases ≥1 entries’ probabilities multiplied by the factor $k\cdot r_{t}$. For pre-symptomatic agents in $I_{a}$, the daily production of second-generation infections is given by the same rule, but only with multiplicative factor $r_{t}$ instead of $k\cdot r_{t}$. Therefore, the effective reproduction number $R_{t}$ is given by the formula

(1) $$\begin{array}{cc} R_{t} & =\left(k\cdot P_{a}\cdot E\left(\tau_{I_{a},R}\right)+P_{s}\cdot E\left(\tau_{I_{a},I_{s}}\right)\right)\cdot E\left(s.c.\right)\cdot r_{t}\;\\ \; & =\left(0.58\cdot 0.17\cdot 7+0.83\cdot 2.85\right)\cdot 0.71\cdot r_{t}\;\\ \; & =2.17\cdot r_{t}. \end{array}$$

where E(s.c.) stands for the mean value of the secondary cases provided by the offsping distribution. Note that no special interpretation is given for the factor $r_{t}$ or the scaling factor 2.17.

## 3. Evolution Process, Particle Filter, and Main Results

The observation data for compartments *T*, *R*, and *D* (colored in yellow in Figure 2) provided by health authorities for Tokyo start on 6 March 2020. However, the epidemic had already started in January at the latest, since the departure of the Diamond Princess from Yokohama took place on 20 January 2020. For this reason, and after several trials, we fixed the following initial conditions for our experiment: 17 January 2020 (long after having chosen this date as the most suitable one for the start of our simulations, we were informed that the first case of COVID-19 detected in Japan was on 16 January 2020). This date corresponds to 49 days before the start of the observations available for Tokyo.

On a daily basis, some parameters have a certain freedom to change their values. In order to describe their evolutions, let us use the notation TN(μ,σ;a,b) for the truncated normal distribution of mean μ, variance $\sigma^{2}$, minimum value *a* and maximal value *b*. For the minimum value *a* and the maximum value *b* of this distribution, we use some prior information from the literature, or the normalized interval [0,1]. For the standard deviation σ, we chose a value corresponding to (b−a)/20. Indeed, it was observed that this value provides enough freedom to the system for its daily evolution, while a larger value generates a spurious self-averaging effect. For the evolution of $r_{t}$ with t≥1, we randomly chose its value according to the distribution $\mathrm{TN}(r_{t-1},0.05;\;0,1)$. The evolution of $P_{d}$ is also given by using a truncated normal distribution, namely, for t≥1 the value $P_{d}\left(t\right)$ was chosen randomly according to the distribution $\mathrm{TN}(P_{d}\left(t-1\right),0.0025;\;0,0.05)$.

Since the time spent undergoing treatment, $\tau_{T}$, is integer valued, its evolution is slightly more complicated, but nevertheless follows a scheme similar to the previous two parameters. The mean value $E(\tau_{T}\left(t\right))$ of $\tau_{T}\left(t\right)$ was chosen randomly according to the distribution $\mathrm{TN}(E\left(\tau_{T}\left(t-1\right)\right),0.75;\;4,19)$, where the minimum and maximum values were fixed according to information provided by health authorities [39]. Then, one constructs a distribution supported on the greatest integer less than or equal to $E(\tau_{T}\left(t\right))$ and the least integer greater than or equal to $E(\tau_{T}\left(t\right))$, and such that its expectation is equal to $E(\tau_{T}\left(t\right))$. The agents entering the compartment *T* on that day are then assigned a time in *T* chosen at random according to this distribution.

Each agent in the compartment $I_{a}$ will infect susceptible agents belonging to the compartment *S* on a daily basis. We now describe this process. Let us denote by

$$\mathrm{MN}\left(x_{j},n;\;p_{j}\right)\equiv \mathrm{MN}\left(0,1,2,3,4,5,n;\;p_{0},p_{1},p_{2},p_{3},p_{4},p_{5}\right),$$

the multinomial distribution for *n* trials in the set of values $x_{j}\in \left\{0,1,2,3,4,5\right\}$ with the probability of $x_{j}$ given by $p_{j}$. We also denote by the vector $X=(X_{0},X_{1},X_{2},X_{3},X_{4},X_{5})$ one realization of this distribution. For example, if $p_{j}$ is the the probability of having *j* offsprings as given in Figure 4, then one has $\sum_{j=0}^{5}X_{j}=n$, and the expectation value for $X_{j}$ is $n\cdot p_{j}$ for any j∈{0,1,2,3,4,5}.

On a given day *t*, assume that the compartment $I_{a}$ contains *n* asymptomatic agents and *m* pre-symptomatic agents. Then, the *n* agents will infect $\sum_{j=1}^{n}j\cdot X_{j}$ susceptible agents, where *X* is one realization of the multinomial distribution $\mathrm{MN}(x_{j},n;\;p_{j}^{a})$, with $p_{j}^{a}=k\cdot r_{t}\cdot p_{j}$ for j∈{1,2,3,4,5} and $p_{0}^{a}=\left(1-k\cdot r_{t}\right)+k\cdot r_{t}\cdot p_{0}$. Similarly, the *m* agents will infect $\sum_{j=1}^{n}j\cdot Y_{j}$ susceptible agents, where *Y* is one realization of the multinomial distribution $\mathrm{MN}(x_{j},m;\;p_{j}^{s})$, with $p_{j}^{s}=r_{t}\cdot p_{j}$ for j∈{1,2,3,4,5} and $p_{0}^{s}=\left(1-r_{t}\right)+r_{t}\cdot p_{0}$.

As highlighted in Figure 2, three compartments, *T*, *D*, and *R*, are associated with observations (collected from [42]) and their values on day *t* are denoted by T(t), D(t) and R(t), respectively. Some uncertainties surround these observations, and these uncertainties are commonly called observation error and will be denoted by σ. For our experiments, we consider two of these observation errors constant over time, while one will be time dependent. In fact, since the daily variations of *T* are quite important, the corresponding uncertainties will take them into account. More precisely, if we write T(t) for the number of agents undergoing treatment at time *t*, we set

$$\begin{array}{ccc} \sigma_{T}\left(t\right) & = & \sqrt{{\left(0.3T(t)+4|T(t)-T(t-1)|\right)}^{2}+400},\;\\ \sigma_{R} & = & 2000,\;\\ \sigma_{D} & = & 100. \end{array}$$

These expressions for the errors have been chosen after several trials; see the remark below. As shown later, even if these values look very large, they lead to a successful selection process.

Let us now describe more precisely the experimental setup. We used a particle filter approach; namely we created a large number *N* of independent simulations (=particles) of the propagation of the epidemic in Tokyo in one experiment. Each of the simulations is a realization of the model, and involves all the daily random processes described above. Typically, we chose *N* = 50 000 or *N* = 100 000, and kept this number constant during each experiment. The initial conditions we used are gathered in Table 3. On a daily basis, a weight *w* was assigned to each particle. Let *i* denote the index of the particle, and let $T_{i}\left(t\right)$, $R_{i}\left(t\right)$, and $D_{i}\left(t\right)$ denote the number of agents in the three colored compartments of Figure 2 for this particle at time *t*. At each time t≥1 one first computes the not normalized weight $W_{i}\left(t\right)$ by the formula

(2) $$W_{i}\left(t\right)≔w_{i}\left(t-1\right)\cdot \exp\left(-\frac{{\left(T_{i}\left(t\right)-T\left(t\right)\right)}^{2}}{2\sigma_{T}{\left(t\right)}^{2}}-\frac{{\left(R_{i}\left(t\right)-R\left(t\right)\right)}^{2}}{2\sigma_{R}^{2}}-\frac{{\left(D_{i}\left(t\right)-D\left(t\right)\right)}^{2}}{2\sigma_{D}^{2}}\right),$$

with the convention that $w_{i}\left(0\right)=\frac{1}{N}$. Then, the normalized weight for the particle *i* is given by

$$w_{i}\left(t\right)≔\frac{W_{i}\left(t\right)}{\sum_{j=1}^{N}W_{j}\left(t\right)}.$$

With these weights, one obtains three distributions for the analyzed values of *T*, *R*, and *D* at time *t*, respectively.

**Remark** **1.** *As already mentioned, the expressions for $\sigma_{X}$ with X∈{T,R,D} were chosen after several trials. Based on the expression *(2)* for the weight, let us provide more information about these observation errors. Clearly, if $\sigma_{X}$ is too big, the factor $\exp\{-\frac{{\left(X_{i}\left(r\right)-X\left(t\right)\right)}^{2}}{2\sigma_{X}^{2}}\}$ will be close to 1 for all particles, and this factor will not play any role. On the other hand, if $\sigma_{X}$ is too small, then only the particles having $|X_{i}\left(t\right)-X\left(t\right)|≅\sigma_{X}$ will keep this factor in (0.1,1), while those having a difference $|X_{i}\left(t\right)-X\left(t\right)|$ much bigger than $\sigma_{X}$ will be assigned an extremely small weight. In addition, since the non-normalized weight *(2)* is defined by the product of three factors, their relative importance is also encoded in the values given to $\sigma_{T}$, $\sigma_{R}$ and $\sigma_{D}$. During the experiments, we observed that the data about the treated agents are clearly more important than the data related to the recovered agents and to the deceased agents for the computation of the effective reproduction number. The choice of two large and constant values for $\sigma_{R}$ and $\sigma_{D}$ reflects this observation. On a daily basis, the exact number of agents who recover, or the exact number of deceased agents, did not match too closely with the real data. However, we kept a weight related to these two quantities because the simulations can also not show arbitrary numbers of recovered agents or of deceased agents. The expression for $\sigma_{T}$ takes into accounted the current number of agents in the compartment T and the daily variation, together with a regularization factor $20^{2}$. In this expression, note that none of the numerical values has a very meaningful interpretation, but it is important that the same values are kept for the computation of the weight of all particles.*

Now, if we keep computing weights with the formula described above, it will soon turn out that very few particles will concentrate almost all weights, and that nearly all other particles would end up with negligible weights. For dealing with this problem, a process called resampling was implemented: For a given time *t*, we took one realization *X* of the multinomial distribution $\mathrm{MN}(i,N;\;w_{i}\left(t-1\right))$ with i∈{1,⋯,N}. Then, for a particle indexed *i*, we created $X_{i}$ copies of the particle and removed the original one. Finally, we assigned a weight 1/N to all new particles. Clearly, some particles will appear several times, but their trajectories will diverge due to the randomness involved on a daily basis.

**Figure 5:**
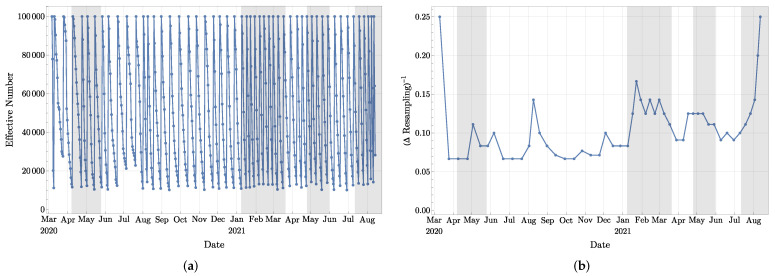
(**a**) The effective number $N_{eff}$ of particles computed with Formula (3). (**b**) The inverse of time between two consecutive resamplings.

One still has to decide when resamplings are organized. For this purpose, let us define the effective number of particles as

(3) $$N_{eff}\left(t\right)≔\frac{1}{\sum_{i=1}^{N}w_{i}{\left(t\right)}^{2}},$$

and observe that if $w_{i}=\frac{1}{N}$ for i∈{1,⋯,N}, then $N_{eff}=N$, while if $w_{i}\approx 1$ for one *i*, and $w_{j}\approx 0$ for all j≠i, then $N_{eff}\approx 1$. For this reason, $N_{eff}$ is often used as an indicator of the number of particles still playing a role in the process. As a consequence, we shall use the following rule: If $N_{eff}<\frac{N}{10}$, then a resampling has to take place. As a practice to stabilize the experiments, a resampling is also performed if the most recent resampling took place 15 days ago. In Figure 5a, we present a typical graph for $N_{eff}$ for a number of particles *N* = 100 000, while in Figure 5b we provide an indication about the time between the resamplings, namely whenever a resampling took place, we set y=1/(numberofdayssincethelastresampling). We shall soon see that this information plays a role in the stability of the predictions.

Let us stress that the computation of the weights and the implementation of the resampling start only on day 50 of the simulation, namely on March 6. Indeed, as there is no observation available before this date, the particles evolve freely and independently.

**Figure 6:**
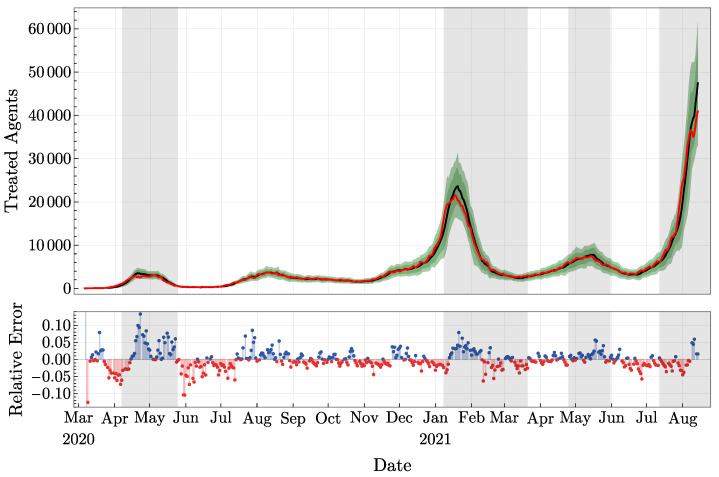
**Upper part**: Treated agents *T*, with the observation values in red and the analyzed mean values in black. 68%, resp. 90%, confidence interval are indicated in green. **Lower part**: Relative difference for the mean values of 1-day forecast and analyzed *T*.

After running the experiment with observation data up to day *t*, we ran the experiment for one more day without observation data. With the weight computed on day *t* still assigned to each particle, one obtains the so-called forecast (or prior) values for $T_{i}$, $R_{i}$, and $D_{i}$, on day t+1. These values generate the corresponding forecast distributions for *T*, *R*, and *D*. Then, by computing the weights $w_{i}\left(t+1\right)$, as explained above, one obtains the analyzed values for the compartments, also called posterior values, and the corresponding analyzed distributions. Clearly, the forecast distributions and the analyzed distributions do not match in general. The relative difference between the mean values of the forecast and the analyzed distributions for *T* can be visualized in Figure 6 (lower part). Let us just emphasize that the weights b computed with three compartments, not only with *T*.

**Figure 7:**
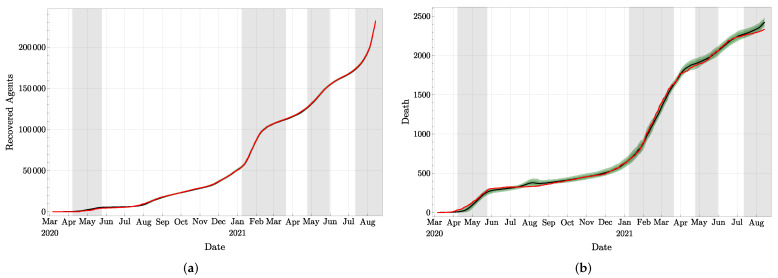
Observation values (in red) and analyzed values with mean value (in black) and 68%, resp. 90%, confidence interval: (**a**) compartment *R* of recovered after treatment and (**b**) compartment *D* of deaths.

**Figure 8:**
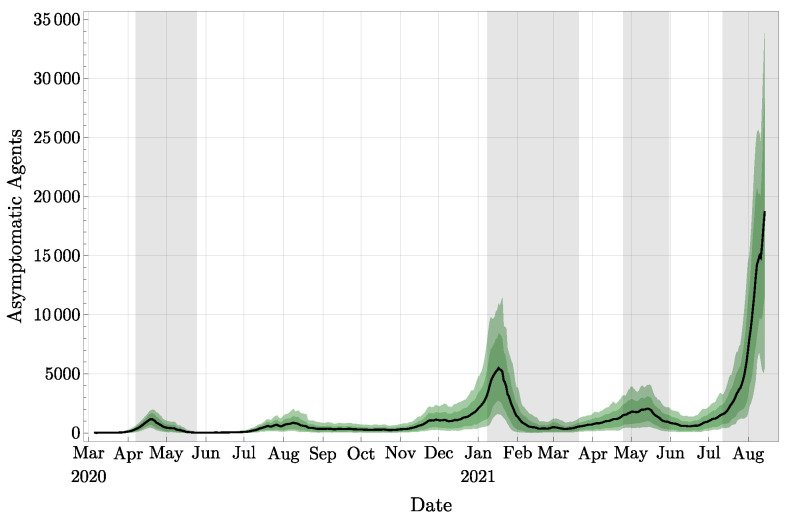
Estimated number of asymptomatic agents in $I_{a}$, with 68% and 90% confidence intervals.

A representation of the three compartments with observations is also presented in Figure 6 (upper part) and in Figure 7. In these plots, the observations are represented in red, and the analyzed distributions are represented in green (confidence intervals) and in black (means). Similarly, we provide in Figure 8 the plot of the total number of asymptomatic agents in $I_{a}$ following the estimate provided in [32]. By the definition of asymptomatic agents, there is no observation corroborating these values.

**Figure 9:**
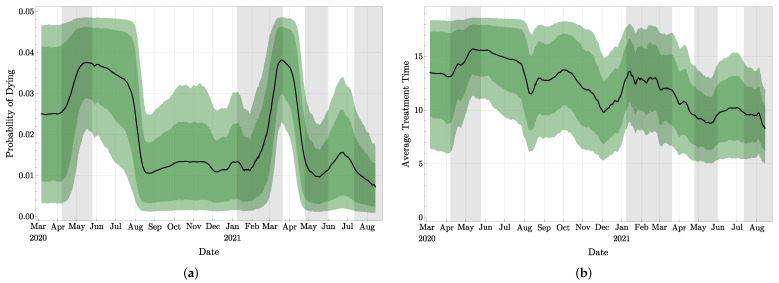
Mean value and 68%, resp. 90%, confidence interval for: (**a**) the probability $P_{d}$ of dying, while in *T* and (**b**) the expected time $E(\tau_{T})$ spent in compartment *T*.

As mentioned in Section 2, $P_{d}$, $P_{r}$, and $E(\tau_{T})$ were evaluated during the experiments, which are the probability of dying, the probability of recovering from *T*, and the average time agents spend in *T*, respectively. With our approach, these quantities are provided with their distributions created by their values taken by the *N* particles. Clearly, if more accurate medical information was available, these parameters could be directly implemented in our model, but in their absence, we have to evaluate them from the observations. For the expected value of $P_{d}$ shown in Figure 9a, observe that the sudden increase visible between mid-February and mid-March 2021 is due to a conjunction of two factors: (1) a large number of death, see Figure 7b, due to the large number of treated agents in January 2021 (as visible in Figure 6) and (2) the relatively small daily number of agents recovering between mid-February and mid-March 2021, as visible in Figure 7a with the small slope of the curve during this period. In other words, this increase in $P_{d}$ is due to the longer stay in compartment *T* of agents who will ultimately die compared to agents who will recover after treatment.

For the expected time $E(\tau_{T})$ spent in *T*, we provide in Figure 9b the distributions $E(\tau_{T})$ deduced from our investigations. The observed long-term trend of decay is expected to be due to the improvement of the medication and treatment for infected patients. Unfortunately, we cannot deduce the difference of time spent in *T* between patients with mild symptoms and patients with serious symptoms from our model.

## 4. Parameters’ Dependence and Stability

**Figure 10:**
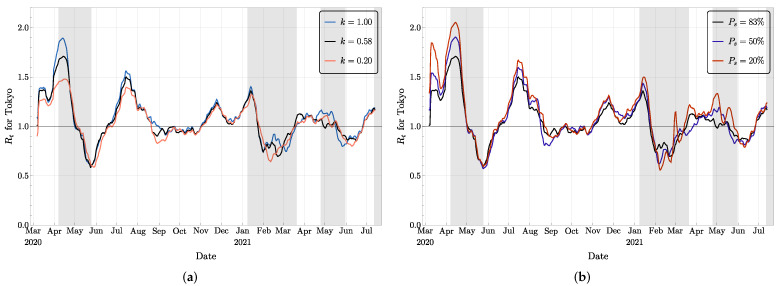
Mean values of $R_{t}$ for: (**a**) different values of the relative infectivity *k* and (**b**) different probability $P_{s}$ of developing symptoms.

As mentioned in Section 2, the relative infectivity *k* of asymptomatic agents compared to pre-symptomatic and symptomatic agents is a very uncertain parameter. For most of our simulations, we used *k* = 0.58 based on the information provided by the literature [32]. Since this value is quite uncertain, we compared the outcomes of simulations with all conditions the same but with *k* = 0.2, 0.58, and 1.0. The corresponding mean values for the effective reproduction number $R_{t}$ are shown in Figure 10a. The patterns are similar, but a larger *k* corresponds to a slightly larger value of $R_{t}$, most of the time. This is not surprising since the factor *k* plays a role in the computation of the effective reproduction number, as shown in (1). On the other hand, with one noticeable exception around late August to early September 2020, the three curves cross the critical value $R_{t}=1$ more or less simultaneously.

Similarly, the probability $P_{s}$ that an infected agent develops symptoms is also a somewhat controversial parameter. For our simulations, we used the proportion of 83% of symptomatic agents and 17% of asymptomatic agents from [32], but other sources mentioned a very different numbers [31]. Since asymptomatic cases are very difficult to detect, and since we cannot be fully confident in these probabilities, a sensitivity test was performed. To do this, we decreased the probability $P_{s}$ to 50% and to 20% and performed the whole experiment while keeping all other parameters the same as the original ones. Compared to the original setting, more agents are asymptomatic and recover without showing any symptoms in these two new scenarios. On the other hand, the number of symptomatic agents is more or less constant since they are dominated by the number of agents undergoing treatment, which is compared and adjusted according to the observations on a daily basis. In Figure 10b, the different curves for the mean $R_{t}$ have similar patterns. However, we observed that a bigger proportion of asymptomatic agents leads to a slightly bigger $R_{t}$. Indeed, more total infected agents have to be created in this scheme, which means that a larger $R_{t}$ is necessary.

**Figure 11:**
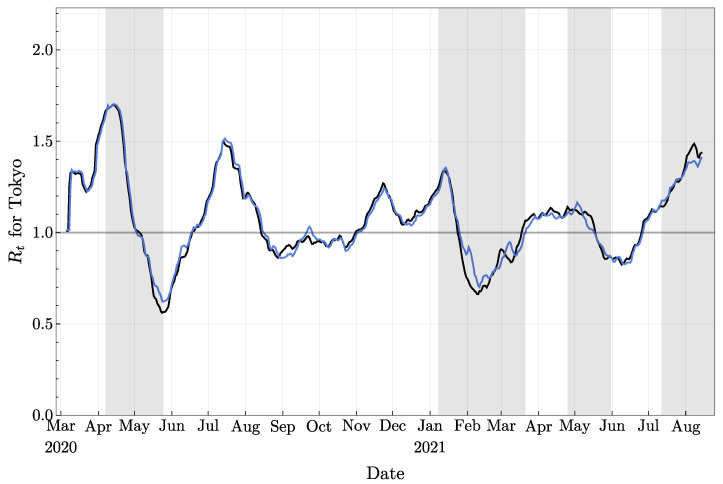
The mean values of the effective reproduction number obtained with two independent experiments, each involving 100 000 particles and the same setting.

Let us mention one observation linking the stability of the computation of $R_{t}$ with the frequency of resamplings. In Figure 11, we provide the mean value of $R_{t}$ for two independent experiments under exactly the same setting, and each of them involves 100 000 particles. Clearly, these two curves are very similar, but at a few places, small differences are visible, such as, for example, in September 2020, in February and first half of March 2021, and in August 2021. Note that the same kind of discrepancies can also be observed in Figure 10 (around the first two mentioned periods). Now, if we look back to Figure 5b, it can be seen that these three periods follow or coincide with periods of time when resamplings took place more frequently (local maxima of the curve presented in Figure 5b). Our understanding is the following: when abrupt changes in the observations are taking place, and/or when the model is not accurate enough, more resamplings are necessary to keep the experiment following the observation data since only a small portion of particles will have the correct behavior. As the weights concentrate on those particles, $N_{eff}$ drops rapidly, and more resamplings are triggered as a consequence. This process also quickly eliminates the short-term diversity of particles: since most of the particles are sampled from that small portion of particles, they will share similar behavior for a few days. This could lead to the following two consequences in the short term: (1) the system is less capable of adapting to further rapid changes and (2) the randomness plays a more important role.

## 5. Discussion and Conclusions

The effective reproduction number $R_{t}$ is certainly one of the most important parameters for the study of an epidemic, but it is also one of the most difficult parameters to estimate. Indeed, because of the delay between the infection of an agent and the appearance of symptoms, the effect of the current $R_{t}$ will only be visible in a few days. Accordingly, the current situation (number of agents in $I_{a}$, $I_{s}$, or in *T*) is related to some effective reproduction numbers which took place over the preceding days. Additionally, since the infectious period lasts for several days, it is not possible (as for example mentioned in [6]) to shift $R_{t}$ by some days to obtain a better picture. These drawbacks are well-known, and are taken into account by medical institutions. Our approach does not solve these problems, but it provides an alternative way of estimating $R_{t}$, and makes it clear that $R_{t}$ is really the reproduction number taking place on day *t*. As emphasized in [6], this synchronicity is not shared by all methods estimating $R_{t}$.

**Figure 12:**
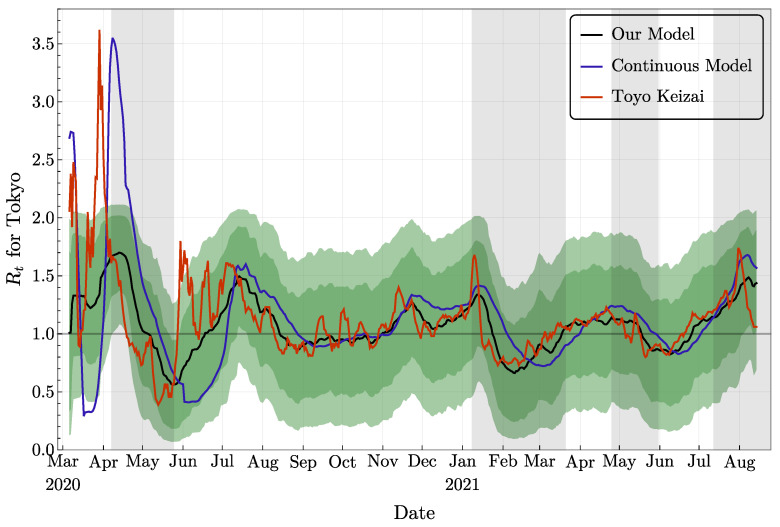
The effective reproduction number for Tokyo computed with different approaches: the black curve and the green confidence intervals are from our approach, the blue curve is provided by [44], and the red curve is borrowed from [42].

In Figure 12, we compare our evaluation for $R_{t}$ with two other resources. One of them is provided by Toyo Keizai, a book and magazine publisher based in Tokyo [42]. Their approach for the computation of $R_{t}$ is completely different from ours, and is based on a simplified version of a formula proposed by H. Nishiura [33]. The second resource is a companion paper [44] in which similar investigations were performed with a different approach: a continuous model (with differential equations) is used for the simulation, and the data assimilation part is based on the ensemble Kalman filter. Clearly, in the first several months of the epidemic, these different methods led to rather diverse values of $R_{t}$. This phenomenon is expected to be due to inaccurate data, irregular release from *T*, and small numbers of agents. On the other hand, since mid-July 2020, the two curves from other studies and our mean $R_{t}$ share very similar patterns. From the figure, one can notice that our approach and the approach of [44] both provide a more stable $R_{t}$ estimation than the approach of [42]. Indeed, Ref. [42] uses the observations for the computation of $R_{t}$ directly, while our approach and the approach of [44] model that pandemic and use data assimilation to produce $R_{t}$ estimations. As a result, the rapid variations in $R_{t}$ estimations do not appear anymore.

**Figure 13:**
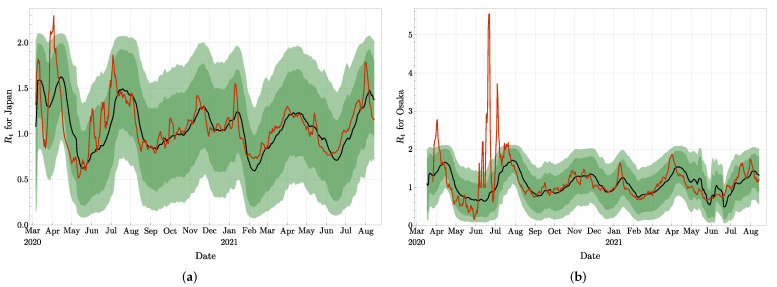
The effective reproduction number for Japan (**a**) and for Osaka (**b**): The black curve and the green confidence intervals are from our approach, and the red curve is borrowed from [42].

**Figure 14:**
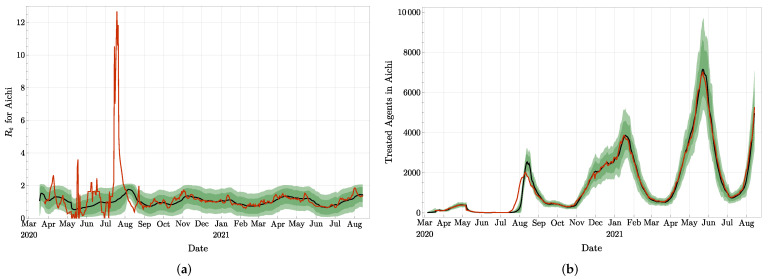
(**a**) Effective reproduction number for Aichi (**a**): The black curve and the green confidence intervals are from our approach, and the red curve is borrowed from [42]. (**b**) Observation value of *T* (in red) and analyzed values with mean value (in black) and 68%, resp. 90%, confidence interval for Aichi.

In Figure 13 and Figure 14a, we present the effective reproduction numbers evaluated for Japan, Osaka, and Aichi. As of September 2021, Japan has about four times more accumulated infected agents than Tokyo, while Osaka has about half of Tokyo, and Aichi about one quarter of Tokyo. Our evaluations are compared once again with the $R_{t}$ provided by [42] and based on a maximum likelihood estimation. It clearly appears on these figures that the methods lead to similar trends and values for $R_{t}$, especially after July 2020, and independently of the size of the epidemic. As for Tokyo, our curves do not present the rapid oscillations, and look more stable.

The previous comparisons with populations of different sizes allows us to emphasize a specific aspect of our approach: the size of the population in *S* does not play any role. In fact, we never introduced the sizes of Tokyo, Osaka, Aichi, or Japan in our settings. The only ingredients which matter for the computation of the effective reproduction number $R_{t}$ are the data corresponding to the three yellow compartments of Figure 2. As a result of this independence of the size of the underlying population, births and deaths (not due to COVID-19) do not play any role. Accordingly, the immunization status of agents in *R* and the vaccination status of the population in *S* are not relevant for our investigations. On the other hand, it is clear that an efficient vaccination campaign, or a change in the variant of the virus, will have an impact in the effective reproduction number, but our approach based on real data automatically takes these parameters into account. In this sense, our model really computes the effective or phenomenological reproduction number.

We believe that our agent-based model, together with the particle filter approach, constitutes a rather simple method for studying the evolution of epidemics. Based on a model for the propagation of the disease, it allows us to perform several tests about uncertain parameters, as shown in Section 4. Note, however, that it implicitly takes some assumptions into account, such as, for example, that only agents in $I_{a}$ can infect new agents. The complexity of the model could also be enlarged, for example, by increasing the number of compartments and flows between them. Another simplification in our agent-based model is the absence of graph structure. Indeed, we could have considered each agent located on a node of a large graph (for example, homogeneous, random, or temporal) and implemented the interactions between agents by the edges of the graph. Such additional structures would have increased the complexity of the model, but the approach with a particle filter could still be implemented. Interacting populations could also be considered: In Figure 14b, we suspect that the sudden increase in infected agents and then agents undergoing treatment, visible around August 2020 in Aichi prefecture, could have been triggered by an influx of infected agents coming from other prefectures. Our model could then be elaborated on a modular network, such as, for example, the one developed in [45]. We plan to work on these issues in the future.

## Figures and Tables

**Table 1:** Probabilities between compartments.

Name	Path	Probability of	Value (%)	Source
$P_{s}$	$I_{a}\to I_{s}$	being symptomatic	83	[32]
$P_{a}$	$I_{a}\to R$	being asymptomatic	17	[32]
$P_{t}$	$I_{s}\to T$	contacting health authorities	78	[36]
$P_{q}$	$I_{s}\to R$	self-quarantining	22	[36]
$P_{d}$	T→D	dying	Evaluated with observations	
$P_{r}$	T→R	recovering after treatment	Evaluated with observations	

**Table 2:** Time spent in compartments (Cpt).

Cpt	Name	Description	Value	Source
*E*	$\tau_{E}$	Latency period	3 days	[37]
$I_{a}$	$\tau_{I_{a},I_{s}}$	Infectious period for pre-symptomatic	Given in Figure 3	[38]
	$\tau_{I_{a},R}$	Infectious period for asymptomatic	Given in Figure 3	
$I_{s}$	$\tau_{I_{s},T}$	Time before treatment for symptomatic	Given in Figure 3	[39]
	$\tau_{I_{s},R}$	Time before recovery for asymptomatic	Irrelevant for the model	
*T*	$\tau_{T}$	Time spent undergoing treatment	Evaluated with observations	

**Table 3:** Initial conditions.

Description	Initial Values
Initial date	17 January 2020
Initial number of infected agents	Uniformly at random in {3,4,5,6,7}
$r_{0}$	Uniformly at random in [0,1]
$\tau_{T}\left(0\right)$	15 days, initial guess motivated by [43]
$P_{d}\left(0\right)$	Uniformly at random in [0,0.05]

## Data Availability

Data available on [42]. Program available upon request.

## References

[1] Cori A., Ferguson N.M., Fraser C., Cauchemez S. (2013). A new framework and software to estimate time varying reproduction numbers during epidemics. Am. J. Epidemiol..

[2] Diekmann O., Heesterbeek J.A.P., Metz J.A.J. (1990). On the definition and the computation of the basic reproduction ratio *R*_0_ in models for infectious diseases in heterogeneous populations. J. Math. Biol..

[3] van den Driessche P., Watmough J. (2002). Reproduction numbers and sub-threshold endemic equilibria for compartmental models of disease transmission. Math. Biosci..

[4] Hethcote H.W. (2000). The mathematics of infectious diseases. SIAM Rev..

[5] Nishiura H., Chowell G., Chowell G., Hyman J.M., Bettencourt L.M.A., Castillo-Chavez C. (2009). The effective reproduction number as a prelude to statistical estimation of time-dependent epidemic trends. Mathematical and Statistical Estimation Approaches in Epidemiology.

[6] Gostic K.M., McGough L., Baskerville E.B., Abbott S., Joshi K., Tedijanto C., Kahn R., Niehus R., Hay J.A., De Salazar P.M. (2020). Practical considerations for measuring the effective reproductive number, *R*_t_. PLoS Comput. Biol..

[7] Páez G.N., Cerón J.F., Cortés S., Quiroz A.J., Zea J.F., Franco C., Cruz É., Vargas G., Castañeda C. (2021). Alternative Strategies for the Estimation of a Disease’s Basic Reproduction Number: A Model-Agnostic Study. Bull. Math. Biol..

[8] Rhodes C.J., Hollingsworth T.D. (2009). Variational data assimilation with epidemic models. J. Theor. Biol..

[9] Van Wees J.-D., Osinga S., van der Kuip M., Tanck M.W.T., Tutu-van Furth A.M. (2020). Forecasting hospitalization and ICU rates of the COVID-19 outbreak: An efficient SEIR model. Bull. World Health Organ..

[10] Aslam M. (2020). Using the kalman filter with Arima for the COVID-19 pandemic dataset of Pakistan. Data Brief.

[11] Zeng X., Ghanem R. (2020). Dynamics identification and forecasting of COVID-19 by switching Kalman filters. Comput. Mech..

[12] Hasan A., Putri E.R.M., Susanto H., Nuraini N. (2021). Data-driven modeling and forecasting of COVID-19 outbreak for public policy making. ISA Trans..

[13] Singh K.K., Kumar S., Dixit P., Bajpai M.K. (2021). Kalman filter based short term prediction model for COVID-19 spread. Appl. Intell..

[14] Evensen G., Amezcua J., Bocquet M., Carrassi A., Farchi A., Fowler A., Houtekamer P.L., Jones C.K., de Moraes R.J., Pulido M. (2021). An international initiative of predicting the SARS-CoV-2 pandemic using ensemble data assimilation. Am. Inst. Math. Sci..

[15] Ghostine R., Gharamti M., Hassrouny S., Hoteit I. (2021). An Extended SEIR Model with Vaccination for Forecasting the COVID-19 Pandemic in Saudi Arabia Using an Ensemble Kalman Filter. Mathematics.

[16] Cheng S., Pain C.C., Guo Y.-K., Arcucci R. (2021). Real-time updating of dynamic social networks for COVID-19 vaccination strategies. arXiv.

[17] Arroyo-Marioli F., Bullano F., Kucinskas S., Rondón-Moreno C. (2021). Tracking R of COVID-19: A new real-time estimation using the Kalman filter. PLoS ONE.

[18] Daza-Torres M.L., Capistrán M.A., Capella A., Christen J.A. (2021). Bayesian sequential data assimilation for COVID-19 forecasting. arXiv.

[19] Armstrong E., Runge M., Gerardin J. (2021). Identifying the measurements required to estimate rates of COVID-19 transmission, infection, and detection, using variational data assimilation. Infect. Dis. Model..

[20] Engbert R., Rabe M.M., Kliegl R., Reich S. (2021). Sequential Data Assimilation of the Stochastic SEIR Epidemic Model for Regional COVID-19 Dynamics. Bull. Math. Biol..

[21] Li X., Zhao Z., Liu F. (2020). Big data assimilation to improve the predictability of COVID-19. Geogr. Sustain..

[22] Mitchell L., Arnold A. (2021). Analyzing the effects of observation function selection in ensemble Kalman filtering for epidemic models. Math. Biosci..

[23] Nadler P., Wang S., Arcucci R., Yang X., Guo Y. (2020). An epidemiological modelling approach for COVID-19 via data assimilation. Eur. J. Epidemiol..

[24] Rebollo T.C., Coronil D. (2020). Predictive data assimilation through Reduced Order Modeling for epidemics with data uncertainty. arXiv.

[25] Silva V.L.S., Heaney C.E., Li Y., Pain C.C. (2021). Data assimilation predictive GAN (DA-PredGAN): Applied to determine the spread of COVID-19. arXiv.

[26] Gómez S., Arenas A., Borge-Holthoefer J., Meloni S., Moreno Y. (2010). Discrete-time Markov chain approach to contact-based disease spreading in complex networks. Europhys. Lett..

[27] Schaum A., Bernal-Jaquez R., Ramos L.A. (2022). Data-assimilation and state estimation for contact-based spreading processes using the ensemble kalman filter: Application to COVID-19. Chaos Solitons Fractals.

[28] Pijpers F.P. (2021). A non-parametric method for determining epidemiological reproduction numbers. J. Math. Biol..

[29] Thompson R.N., Stockwin J.E., van Gaalen R.D., Polonsky J.A., Kamvar Z.N., Demarsh P.A., Dahlqwist E., Li S., Miguel E., Jombart T. (2019). Improved inference of time-varying reproduction numbers during infectious disease outbreaks. Epidemics.

[30] Buitrago-Garcia D., Egli-Gany D., Counotte M.J., Hossmann S., Imeri H., Ipekci A.M., Salanti G., Low N. (2020). Occurrence and transmission potential of asymptomatic and presymptomatic SARS-CoV-2 infections: A living systematic review and meta-analysis. PLoS Med..

[31] He Z., Ren L., Yang J., Guo L., Feng L., Ma C., Wang X., Leng Z., Tong X., Zhou W. (2021). Seroprevalence and humoral immune durability of anti-SARS-CoV-2 antibodies in Wuhan, China: A longitudinal, population-level, cross-sectional study. Lancet.

[32] Byambasuren O., Cardona M., Bell K., Clark J., McLaws M.-L., Glasziou P. (2020). Estimating the extent of asymptomatic COVID-19 and its potential for community transmission: Systematic review and meta-analysis. Off. J. Assoc. Med. Microbiol. Infect. Dis. Can..

[33] Nishiura H. Real-Time Estimation of the Effective Reproduction Number of COVID-19 in Japan. https://github.com/contactmodel/COVID19-Japan-Reff.

[34] Buonomo B., Marca R.D., d’Onofrio A., Groppi M. (2022). A behavioural modelling approach to assess the impact of COVID–19 vaccine hesitancy. J. Theor. Biol..

[35] Gatto M., Bertuzzo E., Mari L., Miccoli S., Carraro L., Casagrandi R., Rinaldo A. (2020). Spread and dynamics of the COVID-19 epidemic in Italy: Effects of emergency containment measures. Proc. Natl. Acad. Sci. USA.

[36] Osaka Prefecture Government Citizens Awareness and Behavior Change of Measures against COVID-19. http://www.pref.osaka.lg.jp/hodo/attach/hodo-40479_4.pdf.

[37] Li R., Pei S., Chen B., Song Y., Zhang T., Yang W., Shaman J. (2020). Substantial undocumented infection facilitates the rapid dissemination of novel coronavirus (SARS-CoV-2). Science.

[38] Pollock A.M., Lancaster J. (2020). Asymptomatic transmission of COVID-19. BMJ.

[39] m3. https://www.m3.com/open/iryoIshin/article/849820/.

[40] World Health Organization (WHO) Transmission of SARS-CoV-2: Implications for Infection Prevention Precautions. https://www.who.int/news-room/commentaries/detail/transmission-of-sars-cov-2-implications-for-infection-prevention-precautions.

[41] Xu X.-K., Liu X.F., Wu Y., Ali S.T., Du Z., Bosetti P., Lau E.H.Y., Cowling B.J., Wang L. (2020). Reconstruction of Transmission Pairs for Novel Coronavirus Disease 2019 (COVID-19) in Mainland China: Estimation of Superspreading Events, Serial Interval, and Hazard of Infection. Clin. Infect. Dis..

[42] Toyo Keizai. https://toyokeizai.net/sp/visual/tko/covid19/en.html.

[43] Tokyo Metropolitan Government. https://www.bousai.metro.tokyo.lg.jp/_res/projects/default_project/_page_/001/010/030/2020080608.pdf.

[44] Sun Q., Richard S., Miyoshi T. (2021). Analysis of COVID-19 in Japan with Extended SEIR model and ensemble Kalman filter. arXiv.

[45] Valdez L.D., Braunstein L.A., Havlin S. (2020). Epidemic spreading on modular networks: The fear to declare a pandemic. Phys. Rev. E.

